# Exploring the Knowledge and Attitude of the Taxi Drivers in the Field of Traffic Rules and Regulations

**DOI:** 10.1155/2022/5280857

**Published:** 2022-11-10

**Authors:** Ali Khani Jeihooni, Tayebeh Rakhshani, Arash Keshavarzi, Seyyed Mansour Kashfi, Mohammad Frarouei, Amirhossein Kamyab

**Affiliations:** ^1^Nutrition Research Center, Department of Public Health, School of Health, Shiraz University of Medical Sciences, Shiraz, Iran; ^2^Department of Public Health, School of Health, Shiraz University of Medical Sciences, Shiraz, Iran; ^3^Department of Epidemiology, School of Health, Shiraz University of Medical Sciences, Shiraz, Iran; ^4^Department of Community Medicine, School of Medicine, Fasa University of Medical Sciences, Fasa, Iran

## Abstract

**Background:**

In all countries, the knowledge of driving guidelines is the most important and critical mechanism to ensure the safety of drivers. Naturally, it is expected that more knowledge and attitude towards driving laws would result in fewer driving violations. The aim of this research is to investigate both the knowledge and attitude of taxi drivers in the field of traffic rules and regulations in Shiraz city, Fars province, in 2019.

**Methods:**

This research is a cross-sectional study, based on which about 1077 taxi drivers were randomly chosen in Shiraz city, Iran. The data collection tool is a demographic information questionnaire and a questionnaire on driving guidance. After collecting these questionnaires, the obtained data were entered into SPSS version 20 and then analyzed by descriptive analyses (mean, standard deviation, and percentage). Afterward, the independent *t*-test, one-way ANOVA, and Pearson correlation coefficient are implemented and investigated.

**Results:**

The mean and standard deviation of knowledge score in drivers were about 26.8 ± 55.2 while the mean and standard deviation of drivers' attitude were about 98.16 ± 59.3. The results showed that there was a significant relationship between the variables studied (*R* = −0.07; *P*=0.02) and drivers' attitudes toward driving (*P*=0.01).

**Conclusion:**

The results of this research show that the mean scores of knowledge and attitude of taxi drivers in Shiraz city are moderate in terms of driving rules and regulations; moreover, among taxi drivers of increasing age, the average score of knowledge was lower.

## 1. Introduction

Traffic accidents are considered a major public health problem in many parts of the world. Yearly, more than 27.1 million people die due to road accidents while 50 million others are injured [1]. According to the World Health Organization (WHO), if serious action is not taken to reduce traffic accidents by 2020, the resulting deaths will increase by up to 67% [2]. In addition, it is expected that road accidents will be the second leading cause of death in countries with low and middle incomes and the third cause of death in high-income countries by 2020 [3]. In Iran, annual traffic accidents account for about 32 cases per 100,000 people, which is the second cause of death, the first cause of lost years of life due to premature death, and the most common cause of injury [4, 5]. Among three general categories of causes of road accidents, including human, environmental, and technical factors, the human factor plays a more prominent role in accidents [6, 7]. Holbert considers 80 to 90% of the causes of accidents to be human factors [8]. In Iran, some factors play in road accidents, such as human errors of 65%, the technical failure of the automobile by 15%, roadside problems by 13%, and weather conditions by 7% [9, 10].

The studies carried out on the incidents are divided into three phases: the first phase is the preincident phase, the second phase is the incident phase, and the third-phase studies is the postaccident phase [11]. The preincident phase studies investigate the drivers' characteristics such as knowledge, attitudes, behaviors, and experiences, and as these studies are preaccidental phase studies, they are very important before the accident and can play a critical role [11]. The incident phase investigates the speed of the vehicles and car and road safety interventions [12], while the postaccident phase assesses the availability of the first aid services to the accident victims [13]. Obviously, by promoting both drivers' and healthcare policymakers' knowledge and attitude levels, we will see fewer traffic accident casualties in the future.

The driver's unsafe behavior is an act that is contrary to the law and driving regulations and affects the level of safety of the driver [14]. The most important reasons for a driver's unsafe behavior are people who are not familiar with safe behaviors (knowledge), they do not feel the need for safe action (attitude), and finally, people are not familiar with how safe behaviors are practiced (performance) [14]. According to several previous studies, drivers, especially taxi drivers in Iran, have insufficient knowledge and less positive attitudes toward driving [15, 16]. Here, it should be mentioned that individual differences such as risk perception, knowledge, and attitude towards the safety of traffic and personality aspects of driving affect safety [17].

Based on studies that have been done, the risk-taking person can be predicted by increasing the level of knowledge of drivers and changing the attitudes of people who change their behavior because their personality is fixed while their attitude is changeable [18]. In all countries, an awareness of driving guidance laws is the most important mechanism to ensure the safety of drivers, and it is naturally expected that the high knowledge of driving rules would result in fewer driving abuses. However, in fact, the rate of driving abuse among the drivers is high. Moreover, the level of awareness of driving and driving laws is at a moderate level.

The knowledge of driving guidance rules has a significant and negative effect on driving offenses. In other words, by increasing awareness of driving guidance rules, the level of driving offenses decreases [19]. Traffic regulations are a form of official norm accepted by the community, in which the main aim is the establishment of order, general welfare, and comfort. Nevertheless, the legislator has taken punitive measures to prevent any violation of these laws. However, the regulatory authority, the police, cannot control the discipline of traffic violations alone. This is because, firstly, it does not allow financial and time costs. Secondly, the proportion of police forces to citizens is not as high as necessary for order in all places. Therefore, the police have to move toward less costly and efficient methods derived from cultural resources and resources derived from the context of society. While being a source of social monitoring and supervision from within, the fine was also imposed by the community and conscience of individuals, permanent and consistent.

Regarding the discussed issues and the announced statistics, the best and most cost-effective way to reduce the occurrence of such incidents is to arrange educational and preventive strategies and plans [20, 21]. Indeed, by providing the statistics and results of previous research, the knowledge, attitudes, and lifestyles of the people of a society have changed. This study investigates the knowledge and attitude of taxi drivers in the field of rules and driving directions.

## 2. Materials and Methods

### 2.1. Study Design and Participants

This cross-sectional study was conducted in Fars province, Shiraz, Iran, in 2019. The population of this study was 1077 taxi drivers working in Shiraz.

### 2.2. Inclusion and Exclusion Criteria

The statistical population of the criteria for entering the study was consenting to participate in the research project and the criteria for withdrawal from the study, the lack of consent of the company in the research project at any time from the research, and incomplete questionnaires.

### 2.3. Sampling Method

The sampling method was simple random, in which 1077 drivers were selected and then the questionnaires were distributed among them.

### 2.4. The Data Collection Tools

In this study, the data-collecting tool was a demographic questionnaire (age, marital status, driving time, years of passing a certificate, driving experience as a job, accident history, education), and a knowledge and attitude questionnaire on driving rules. Based on similar previous studies [15, 22], the knowledge and attitude questionnaires were used to assess drivers' knowledge and attitude levels. The knowledge questionnaire consisted of 13 questions based on Iran's driver's license examination. The internal consistency of the knowledge questionnaire was assessed by Cronbach's alpha (*α* = 0.82). The attitude questionnaire, which was prepared under the supervision of experienced traffic police officers, included seven questions regarding using seat belts, exceeding speed limits, driving in prohibited areas, keeping a safe distance from the next vehicle, crossing the road centerline, stopping driving before entering a main street from a side street, and eating and drinking while driving. The internal consistency of the attitude questionnaire was assessed by Cronbach's alpha (*α* = 0.76). Scoring this questionnaire for the knowledge part was such that the correct answer was given at score 1, and the wrong answer was 0. The maximum score in this questionnaire was 14, and the minimum score was 0. The attitude section of this questionnaire included seven questions with five options (totally agree to totally disagree). Questions 1, 2, 3, and 5 have reciprocal scores. The minimum score in this questionnaire was 1, and its maximum score was 35. To calculate the content validity ratio using the panel of experts used to measure the content validity index, single items were examined using three spectra: “The item is necessary, the item is useful but not necessary, the item is not necessary.” To determine the reliability of the tool, a questionnaire was distributed among 60 drivers who were eligible for inclusion in the study.

### 2.5. Statistical Analysis

After analyzing by SPSS software, its Cronbach's alpha coefficient for the knowledge level was 0.82 and for the attitude level, was 0.76. The results showed that the data were analyzed by SPSS software version 20, so that the data were normalized by the Kolmogorov–Smirnov test. After confirmation of the normality of the data, an independent *t*-test, one-way ANOVA, and Pearson correlation coefficient were used for data analysis. The significance level in all tests was considered to be 0.05.

## 3. Results and Discussion

### 3.1. Results

In general, 1077 people were included in the study. The average age of these drivers was 32.28 ± 11.34. Of 1077 drivers surveyed, the highest frequency in the age group of over 31–40 years was 32.1% (346), 26–30 years (20.8% (224)), and then the age group was 41–50 years old (17.5% (188 people)). The average years of passing the certificate were 15.22 ± 10.29 and the average driving time in the drivers was 4.93 ± 3.33, and the maximum driving hours were 1–2 hours (31.6) 340, and then 5–10 hours (31.2) was 336. Most of the drivers participating in the married study were 750 (69.6%); the highest level of education was for drivers with a high degree of 394 (36.6%); undergraduate students, 262 (24.3%); and then Cycle 177 (16.4%); see Table 1.

The mean and standard deviation of knowledge scores in drivers was 8.26 ± 2.55 and the mean and standard deviation of attitude in drivers was 16.98 ± 3.59, see Table 2.

The results showed that there was a significant relationship between the variables studied between age and the knowledge variable (*R* = −0.4, *P*=0.14). However, there was no significant relationship between the other variables and the mean score of knowledge. In addition, the results showed that there was no significant relationship between attitude mean scale and quantitative variables with attitude score (see Table 3).

The results of ANOVA showed that there was no significant difference between the studied variables and the mean scores of knowledge and attitude. There was a significant difference between drivers' perceptions of driving and their knowledge (*P*=0.01). The drivers who were driving as their main occupation had a higher level of knowledge than those who saw driving as their second job. The results of the LSD test showed that there was a significant difference between drivers who were driving as their main occupation and drivers who had a second job driving (*P*=0.004) or regular occupation and occasionally (*P*=0.02) (see Table 4).

## 4. Discussion

The personality characteristics of people are constant, but their attitudes and knowledge can be changed, and this can approximately decrease the number of accidents [21]. The aim of this research is to investigate the knowledge and attitude of taxi drivers towards driving laws in Shiraz in 2019.

The results of this study showed that the mean and standard deviation of knowledge score in drivers was 8.26 ± 2.55 and the mean and standard deviation of attitude in drivers was 16.98 ± 3.59. Regarding the minimum and maximum scores in knowledge and attitude, it can be said that the taxi drivers in the city of Shiraz had a moderate level of knowledge and attitude. This finding was in agreement with the results of the studies of Redhwan and Karim in Malaysia [23], al-Dahrani et al. in Saudi Arabia [24], and Shams et al. in Tehran [25]. However, it was not consistent according to the results of Riaz and Shahid [26], and a study by Bachani et al. in Kambia [27]. The reasons for this discrepancy include the sample size, the statistical society, and the type of questionnaires used.

The computational results of this research showed that drivers' age had a significant relationship with the knowledge score, and with increasing age, the average knowledge score of drivers was lower. One of the reasons for this issue is the lack of attention, less attention and safety to drivers, or it could be due to a lack of a periodic driving license test. The results also showed that there was no significant relationship between the knowledge score and other demographic variables. This finding was not consistent with the results of Jelalian et al. [28]. In their study, Jelalian et al. showed that there is a significant relationship between education, marital status, and average knowledge scores [28].

The results of this research showed that there was no significant relationship between age, number of years of driving, average driving time, accident history, education, and marital status, and the driver's view of driving as a job, with a mean score of attitude. This finding was consistent with the results of the study of Tajvar et al. in Iran [15].

Moradi et al. stated in their study that there is a significant relationship between the age and occupation of drivers with their attitudes [15]. The reasons for this discrepancy can be seen in the type of statistical community in the study. In this study, the statistical population was surveyed by taxi drivers, as in the study of motorcycle drivers [15].

The descriptive results of the knowledge questionnaire showed that the predominant drivers of good knowledge and more than average were prohibited from speeding rules, distance from the front car, car park rules and park departures, priority and stopping places. However, the awareness of most drivers to the lights of the guide was low.

The descriptive results of the attitude questionnaire in drivers showed that taxi drivers had a positive attitude toward seat belts, driving speed, passage from the prohibited passage, distance from front cars, nondrinking and driving while driving, and compliance with the rules. This finding was not consistent with the results of the study by Habibi et al. [29]. Habibi et al. in their study stated that the highest frequency of hazardous behavior was related to not wearing a seat belt during the route [29].

### 4.1. Strength and Limitation

One of the strength points of this study is that the data were sufficiently valid as the researcher in SPSS spontaneously, carried out the data at the data entry stage, the existence of the researcher during the collection of information, and described the questions for drivers to understand more questions. In contrast, there are some limitations to this study as follows:

1. No question that the belt was closed during driving.
2. Failure to check the drivers' mental status.
3. Lack of a questionnaire for checking both the behavior and performance of drivers.
4. Cross-sectional study.
5. Lack of extendibility of the results of this study to other points.

## 5. Conclusion

The results of this research showed that the mean scores of knowledge and attitude of taxi drivers in Shiraz were moderate in terms of driving rules and regulations.

The results of the knowledge questionnaire showed that the drivers who had good knowledge, or more than average, were prohibited from speeding rules, distance from the front car, car park rules and park departures, priority and stopping places. However, the awareness of most drivers to the lights of the guide was low.

Furthermore, the results of the attitude questionnaire on drivers showed that taxi drivers had a positive attitude toward seat belts, driving speed, passage from the prohibited passage, distance from front cars, nondrinking and driving while driving, and compliance with the rules.

The computational results of this research showed that with increasing age, the average knowledge score of drivers was lower, which could be due to a lack of a standard periodic testing system for assessing drivers' knowledge.

### 5.1. Policy Recommendations

It is suggested that by conducting periodic tests for drivers in order to assess their levels of knowledge and correct their misunderstandings towards traffic rules, organizing free training courses for drivers periodically, more police monitoring of taxi drivers, and establishing centers under the guidance of the directorate of driving to provide taxi drivers with certificates that are related to knowledge, attitude, and mental health of drivers would enhance the level of both knowledge and attitude of taxi drivers.

## Figures and Tables

**Table 1:** Frequency distribution of demographic variables of drivers participating in the study.

Variable		Number	Frequency (%)
Marital status	Married	750	69.6
	Single	316	29.3
	Dead or separated wife	11	1

Education	Illiterate/elementary	68	6.3
	Cycle	177	16.4
	Diploma	394	36.6
	Assistant	107	9.9
	Masters	262	24.3
	Masters and higher education	69	6.4

Driver as a job	Main job	358	33.2
	second job	234	21.7
	Ordinary driver	485	45

Crash history	Yes	439	40.1
	No	620	56.6

**Table 2:** Determine mean and standard deviation of knowledge and attitude in taxi drivers.

Variable	Number of questions	Maximum-minimum	Average ± standard deviation
Knowledge	13 questions	17–1	8.26 ± 2.55
Attitude	7 questions	35–7	16.98 ± 3.59

**Table 3:** Determining the relationship between variables (age, average years of passage of certificate, and number of driving hours) with mean score of knowledge and attitude.

Variable	Attitude		Knowledge	
	*R*	*P* value^*∗*^	*R*	*P* value^*∗*^
Age	−0.07	0.02	0.04	0.14
The number of years passed since the certificate was issued	0.01	0.76	−0.002	0.94
Average driving hours	−0.04	0.15	0.02	0.41

**Table 4:** Determining the relationship between variables (gender, marital status, education, and accident history) with mean score of knowledge and attitude.

Variable		Knowledge		Attitude	
		Mean ± SD	*P* value^*∗*^	Mean ± SD	*P* value^*∗*^
Crash history	Yes	8.16 ± 2.58	0.13^*∗*^	16.78 ± 3.84	0.12^*∗*^
	No	8.38 ± 2.56		17.13 ± 3.49	

Marital status	Married	8.31 ± 2.53	0.15^*∗∗*^	17.03 ± 3.56	0.65^*∗∗*^
	Single	8.14 ± 2.06		16.5 ± 3.70	
	Dead or separated wife	0		19th	

Education	Illiterate/elementary	8.93 ± 2.31	0.1^*∗∗*^	18.00 ± 2.91	0.38^*∗∗*^
	Cycle	8.53 ± 2.40		16.98 ± 3.62	
	Diploma	8.18 ± 2.54		16.77 ± 3.88	
	Assistant	8.16 ± 2.75		16.98 ± 3.70	
	Masters	8.40 ± 2.40		17.20 ± 3.13	
	Senior and higher	7.64 ± 2.93		17.00 ± 3.20	

Driving situation	Main job	8.56 ± 2.46	0.01^*∗∗*^	17.22 ± 3.75	0.26^*∗∗*^
	second job	7.96 ± 2.65		16.86 ± 3.35	
	Ordinary driver/occasional	8.19 ± 2.52		16.83 ± 3.60	

^*∗*^Independent *t*-test test; ^*∗∗*^One-way ANOVA test.

## Data Availability

The datasets used and/or analyzed during the current study are publicly available from the corresponding author on reasonable request.

## Supplementary Materials

Supplementary Materials The questionnaire, which was used in this study in order to investigate drivers, was added as a supplementary file under the supplementary section.
