# Supplementary material for: Exploring the Knowledge and Attitude of the Taxi Drivers in the Field of Traffic Rules and Regulations
Source: ScientificWorldJournal. 2022 Nov 10;2022:5280857. doi: 10.1155/2022/5280857 (PMC9671739; doi:10.1155/2022/5280857)
Supplement: Supplementary Materials — The questionnaire, which was used in this study in order to investigate drivers, was added as a supplementary file under the supplementary section. [file 5280857.f1.pdf]

Questionnaire investigating drivers' knowledge and attitude in the field of traffic rules and regulations

I. Demographic Information Questions

Driver age:

Marital status:

Years of passing a certificate:

Level of education:

Driving hours per day:

Type of automobile:

Accident history: □ Positive □ Negative

Driving experience as a job:

□ Main job

□ Second job

□ Driving occasionally (not as a job)

II. Questions on Driving Guidance

- Knowledge-related Questions

1. What does a yellow light at an intersection mean?

A. To continue the route if possible
B. To be ready to continue the route
C. To stop behind the stop line
D. If there are no pedestrians crossing, we would continue the route

2. How fast are you allowed to drive while driving in the city?
   A. 30 km/h on local passages and squares
   B. 20 km/h on local passages and squares
   C. 40 km/h on local passages and squares
   D. 50 km/h on local passages and squares

3. What is the maximum driving speed limit on main suburban roads?
   A. 80 km/h during the day and 90 km at night
   B. 95 km/h during the day and 85 km at night
   C. 90 km/h during the day and 80 km at night
   D. 110 km/h during the day and 90 km at night

4. You are driving behind another car on a wet road. How long should the time interval be between you and your front car?
   A. One second
   B. Two seconds
   C. Three seconds
   D. Four seconds

5. What should be done while leaving a car park?
   A. To use the mirrors and turn your head to the left and look back for final control
   B. To sign the other drivers if they should drive slowly
   C. Not to drive backward if another car is not parked in front of your car
   D. You are allowed to sign using your hands

6. How is the right to priority in three ways?
   A. With the automobile located on the wider street
   B. With the automobile that has reached the three way earlier
   C. With the automobile that moves straightforward
   D. With the automobile that moves fast

7. What should be done if a car suddenly comes out of a side street in front of you?
   A. To slow down and get ready to stop
   B. To pass by quickly and blow the horn

C. To pass by the car by steering the car
D. To announce your presence using high light

8. When should the tire pressure control be done?
   A. When the tires are cold
   B. After riding a long distance
   C. After a high-speed drive
   D. When the tires are hot

9. What is the basic driving rule for driving on the freeways?
   A. To use the lane that has the least traffic
   B. To always drive to the right unless you are going to overtake
   C. To overtake from a lane which is clear
   D. To drive faster than 90 km / h.

10. In the “Stop Sign’s” area
    A. You may not stop your automobile in this area except while loading or unloading passengers
    B. You may not stop even for a moment
    C. You may not park at all, but you may stop
    D. It depends on the situation

11. At the intersections, in order to turn to the right
    A. You may turn from the far right lane
    B. You may turn from the far left lane
    C. In order to turn right at intersections, there is no need to use the far right lane
    D. You may turn from the middle lane

12. You are driving the allowed speed. The car behind you wants to overtake. Can you stop him from overtaking?
    A. Not at all
    B. Not unless it is safe
    C. Yes, because the driver is doing something dangerous
    D. Yes, because the driver is breaking the law

13. From the dashed line in the middle of the street
    A. It is only allowed to turn
    B. It is only allowed to overtake
    C. You are allowed to turn, but you are not allowed to overtake
    D. They can be used for turning and overtaking

- Attitude-related Questions

Dear driver, while thanking you for your sincere cooperation in this research, please express your opinion in a realistic way about the following questions:

1. Wearing a seat belt can be a deterrent factor to driving

   □ Completely agree □ Agree □ No comment □ Disagree □ Completely disagree

2. During a light traffic on intercity roads, you may drive faster than allowed

   □ Completely agree □ Agree □ No comment □ Disagree □ Completely disagree

3. In emergencies, you may drive through prohibited passages

   □ Completely agree □ Agree □ No comment □ Disagree □ Completely disagree

4. It is essential to keep your distance from the front car while driving

   □ Completely agree □ Agree □ No comment □ Disagree □ Completely disagree

5. In the very light traffic, you may cross the continuous line in the middle of the road

   □ Completely agree □ Agree □ No comment □ Disagree □ Completely disagree

6. A complete stop is required before entering the main street from the side street

   □ Completely agree □ Agree □ No comment □ Disagree □ Completely disagree

7. Drinking or eating while driving is dangerous in any situation

   □ Completely agree □ Agree □ No comment □ Disagree □ Completely disagree
